# Effect of Omega-3 Supplementation on Self-Regulation in Typically Developing Preschool-Aged Children: Results of the Omega Kid Pilot Study—A Randomised, Double-Blind, Placebo-Controlled Trial

**DOI:** 10.3390/nu13103561

**Published:** 2021-10-12

**Authors:** Lauren A. Roach, Mitchell K. Byrne, Steven J. Howard, Stuart J. Johnstone, Marijka Batterham, Ian M. R. Wright, Anthony D. Okely, Renate H. M. de Groot, Inge S. M. van der Wurff, Alison L. Jones, Barbara J. Meyer

**Affiliations:** 1 School of Medicine, Faculty of Science, Medicine and Health, University of Wollongong, Wollongong, NSW 2522, Australia; lroach@uow.edu.au (L.A.R.); alisonj@uow.edu.au (A.L.J.); 2 Illawarra Health and Medical Research Institute, University of Wollongong, Wollongong, NSW 2522, Australia; ian.wright@jcu.edu.au (I.M.R.W.); tokely@uow.edu.au (A.D.O.); bmeyer@uow.edu.au (B.J.M.); 3 College of Health and Human Sciences, Charles Darwin University, Darwin, NT 0909, Australia; 4 School of Psychology, Faculty of the Arts, Social Sciences and Humanities, University of Wollongong, Wollongong, NSW 2522, Australia; stevenh@uow.edu.au; 5 Early Start, School of Education, Faculty of the Arts, Social Sciences and Humanities, University of Wollongong, Wollongong, NSW 2522, Australia; 6 Brain & Behaviour Research Institute, School of Psychology, Faculty of the Arts, Social Sciences and Humanities, University of Wollongong, Wollongong, NSW 2522, Australia; sjohnsto@uow.edu.au; 7 Statistical Consulting Centre, School of Mathematics and Applied Statistics, Faculty of Engineering and Information Sciences, University of Wollongong, Wollongong, NSW 2522, Australia; marijka@uow.edu.au; 8 College of Medicine and Dentistry, James Cook University, Cairns, QLD 4870, Australia; 9 Early Start, School of Health and Society, Faculty of the Arts, Social Sciences and Humanities, University of Wollongong, Wollongong, NSW 2522, Australia; 10 Conditions for Lifelong Learning, Faculty of Educational Sciences, Open University of the Netherlands, 6419 Heerlen, The Netherlands; renate.degroot@ou.nl (R.H.M.d.G.); inge.vanderwurff@ou.nl (I.S.M.v.d.W.); 11 Fiona Stanley and Fremantle Hospitals Group, South Metropolitan Health Service, Perth, WA 6150, Australia; 12 School of Medicine, Molecular Horizons, Faculty of Science, Medicine and Health, University of Wollongong, Wollongong, NSW 2522, Australia

**Keywords:** n-3 LCPUFAs, self-regulation, preschool-aged children, executive function, HS-Omega-3 Index^®^

## Abstract

Supplementation of omega-3 long chain polyunsaturated fatty acids (n-3 LCPUFA) may enhance self-regulation (SR) and executive functioning (EF) in children of preschool age. The aim of the Omega Kid Study was to investigate the effect of n-3 LCPUFA supplementation on SR and EF in typically developing preschool-aged children. A double-blind placebo-controlled pilot trial was undertaken, the intervention was 12 weeks and consisted of 1.6 g of eicosapentaenoic acid (EPA) and docosahexaenoic acid (DHA) per day compared to placebo. The HS-Omega-3 Index^®^ was assessed by capillary blood samples at baseline and post-intervention. Seventy-eight children were enrolled and randomised to either the n-3 LCPUFA treatment (*n* = 39) or placebo (*n* = 39) group. Post intervention, there was a significant three-fold increase in the HS-Omega-3 Index^®^ in the n-3 LCPUFA group (*p* < 0.001). There were no improvements in SR or EF outcome variables for the n-3 LCPUFA group post intervention compared to the placebo group determined by linear mixed models. At baseline, there were significant modest positive Spearman correlations found between the HS-Omega-3 index^®^ and both behavioural self-regulation and cognitive self-regulation (r = 0.287, *p* = 0.015 and r = 0.242, *p* = 0.015 respectively). Although no treatment effects were found in typically developing children, further research is required to target children with sub-optimal self-regulation who may benefit most from n-3 LCPUFA supplementation.

## 1. Introduction

Omega-3 long chain polyunsaturated fatty acids (n-3 LCPUFA), eicosapentaenoic acid (EPA) and docosahexaenoic acid (DHA) are important nutrients across the lifespan. In utero, n-3 LCPUFA are required for the closing of the neural tube [1], while during infanthood, breastmilk is enriched with DHA [2]. As the child approaches early childhood, n-3 LCPUFA are required for optimal brain function, with EPA of particular benefit due to its anti-inflammatory and vasodilatory properties [3]. Furthermore, the release of EPA from endothelial cells lining blood vessels form eicosanoids, which reduce blood clotting and increase blood flow [4]. This is of benefit to the brain by improving blood flow and integrity of the blood-brain barrier [5].

Across the lifespan, DHA is a major structural component of brain cells and is required for the formation of new neuronal cells and supporting neuronal survival [6]. DHA is found in the phospholipid bilayer of the cell membrane in the brain and contributes to the flexibility and fluidity of the membrane, which in turn improves the function of membrane proteins and neurotransmitters such as dopamine and serotonin [6]. Given the limited ability of humans to synthesise DHA from its precursor alpha-linoleic acid [7], it is preferable to consume pre-formed EPA and DHA rather than rely on the conversion [8]. This is problematic, given that children’s intake of n-3 LCPUFA and their primary food sources (fish and seafood) is generally low [8].

Low intake of n-3 LCPUFA, which is reflected in erythrocyte levels, may have behavioural implications. In early childhood lower erythrocyte n-3 LCPUFA levels have been associated with attention deficit hyperactivity disorder (ADHD) [9,10,11,12,13]. Given that ADHD is associated with deficits in self-regulation and executive functions, sufficient intake of n-3 LCPUFA may be important to support the development of behavioural/cognitive processes of self-regulation and executive functions. Self-regulation is defined by Murray et al. (2015) as “the act of managing cognition and emotion to enable goal-directed actions such as organizing behaviour, controlling impulses, and solving problems constructively” [14]. It is comprised of three domains: cognitive, behavioural, and emotional self-regulation. Executive function is seen as the capacity aspect of cognitive self-regulation, and includes the ability to shift between tasks, inhibition of impulses, and working memory [15].

Neurobiological development during early childhood leads to accelerated changes in self-regulation and executive functions and presents an optimal time to intervene due to the period of rapid brain maturation [14,16]. Any effort to enhance self-regulation and executive functions in early childhood may lead not only to improvements in terms of school readiness but also long-term social and developmental outcomes into early adulthood [17,18]. Greater self-regulation has been associated with better school engagement and academic performance, while poor self-regulation is linked to internalising and externalising problems in primary school [17]. Into adulthood, the long-term impacts of improved self-regulation in early childhood include positive influences on health, wealth and criminal outcomes [18].

Previous n-3 LCPUFA intervention trials for children with ADHD (a condition characterised by poor self-regulation) have shown benefits for working memory, cognition, inattention, hyperactivity and oppositional/defiant behaviour [19,20,21]. These trials are generally conducted in children of primary school age to adolescence (6–12 years) as ADHD is most commonly diagnosed at this time. For children at preschool age, there is a paucity of evidence about the relationship between n-3 LCPUFA supplementation and self-regulation, or more generally behavioural and cognitive measures of executive function. However, there is some reason to expect positive impacts of n-3 LCPUFA supplementation on cognitive development in preschool children, with trials showing beneficial effects on vocabulary, working memory and intelligence scales [22,23,24].

Self-regulation is controlled by the prefrontal cortex [25], and this region of the brain can be measured by electroencephalography (EEG) technology to observe direct neurological processing [26]. The effects of n-3 LCPUFA on preschool-aged children has not been assessed by EEG technology. However, N-3 LCPUFA supplementation has previously resulted in changes to this region of the brain directly measured through functional magnetic resonance imaging, in healthy boys aged 8–10 years, with DHA supplementation for 8 weeks resulting in increased activation of the dorsolateral prefrontal cortex during sustained attention [27]. Showing the importance of having a direct measure of neurological processing in n-3 LCPUFA interventions.

The primary aim of the Omega Kid study was to investigate the effect of n-3 LCPUFA supplementation on self-regulation in preschool-aged children compared to a placebo supplementation. Secondary aims included investigating the effect of n-3 LCPUFA supplementation on executive function, EEG measures and ADHD symptoms.

We hypothesised that: (1) the n-3 LCPUFA supplementation would result in improved self-regulation, and (2) the n-3 LCPUFA supplementation would result in enhanced executive functioning.

## 2. Materials and Methods

### 2.1. Study Design and Participant Criteria

A 12-week randomised, double-blind placebo-controlled pilot study was conducted to investigate the effect of n-3 LCPUFA supplementation on self-regulation compared to a placebo in preschool-aged children. The study was approved by the University of Wollongong Human Research Ethic Committee (2019/171) and was registered with the Australian New Zealand Clinical Trial Registry (ACTRN12619000731190). This manuscript adheres to the CONSORT guidelines for pilot and feasibility trials (Table S1 CONSORT Checklist). The feasibility of the study protocol has been assessed and reported elsewhere [28].

Inclusion criteria included children aged 3 to 5 years of age. Exclusion criteria included: allergies to fish, milk and soybean, blood clotting disorder, on blood thinning medication, upcoming surgery, a known developmental delay (excluding ADHD), and high pre-trial dietary intake of n-3 LCPUFA supplements (with study dose consuming over 3 g/day, [29]).

### 2.2. Recruitment and Randomisation

Children were recruited through university networks, local day care centres and playgroups. Once enrolled in the study children were randomised to either the omega-3 or placebo group as per the parallel study design. Randomisation was completed without stratification using Stata statistical software, in a 1:1 allocation ratio by a biostatistician. The researchers and participants (families and children) remained blinded for the duration of the intervention and throughout data analysis. The study code was kept by a member of the research team who was not involved in data collection and analysis and was only disclosed after primary data analysis was completed. Children who attended the first clinic visit received either tickets to visit a children’s museum or a voucher for the children’s museum gift shop. Children who completed the intervention received a yearly pass to the children’s museum or a gift card to the gift shop of equal value.

### 2.3. Intervention

Details of the intervention have been published [28]. In brief, the intervention was single serve sachets of powder containing either microencapsulated Tuna Oil powder (Driphorm^®^ HiDHA^®^ 50) for the n-3 LCPUFA group or high oleic acid sunflower oil powder for the placebo group. Both treatments were delivered in a base vanilla flavoured powder. The n-3 LCPUFA treatment delivered a total daily dose of 1.6 g EPA and DHA (1.3 g DHA and 0.3 g EPA). Participants were advised to consume one sachet daily with the evening meal mixed into either warm milk or yoghurt. Both the n-3 LCPUFA and placebo sachets were provided by Nu-Mega Ingredients, Australia.

### 2.4. Data Collection

The study involved two clinic visits, at baseline and at 12 weeks post-intervention between September and December 2019. Children attended the University of Wollongong with their parent/caregiver and completed practical assessments while their parent/caregiver completed behavioural questionnaires. Children then provided a finger prick blood sample.

Families were contacted throughout the intervention via email to check if there were any problems and to provide families an opportunity to report any adverse events. In addition, families were encouraged to report adverse events throughout the study.

### 2.5. Outcome Measures

#### 2.5.1. Primary Outcome Measure

The primary outcome of self-regulation was assessed by the Head, Toes, Knees Shoulders (HTKS) task [30] and was completed by the children and administered by a trained research assistant. The HTKS task asks children to remember corresponding body parts and to complete the opposite task when instructed (for example, the child is instructed to touch their toes when they are asked to touch their head). As such, this task requires children to inhibit the impulse to simply perform the action as directed, hold the body part correspondences in mind, and flexibly shift between these correspondences [30]. A score is derived by the number of correct responses by the child, with a higher score indicating better self-regulation.

Self-regulation was also assessed by the Child Self-Regulation and Social Behaviour Questionnaire (CSBQ) [31], completed by parent/caregivers. The CSBQ contains 34-items with responses ranging from 1 to 5, (not true to certainly true) [31]. These responses are then used to derive self-regulation subscales of Cognitive Self-Regulation, Behavioural Self-Regulation, and Emotional Self-Regulation in addition to subscales of Sociability, Prosocial Behaviour, Externalising Problems and Internalising Problems [31].

#### 2.5.2. N-3 LCPUFA Erythrocyte Levels

The HS-Omega-3 Index^®^ was a manipulation check for the intervention. To measure the HS-Omega-3 Index^®^ [32] capillary blood samples were collected at baseline and post-intervention using an Accu-Chek Safe-T-Pro Plus single use lancing device. Samples were stored on a filter paper provided by Omegametrix and were kept at −80 °C until they were shipped and sent on dry ice to Omegametrix, Martinsried, Germany for analysis using standard methodology [33]. To measure whole blood fatty acid concentrations, fatty acids underwent transesterification to methyl esters and were analysed using gas chromatography by a GC2010 Gas Chromatograph (GC) (Shimadzu, Duisburg, Germany) equipped with a SP2560, 100 m column (Supelco, Bellefonte, PA, USA) with hydrogen as the carrier gas. Standard fatty acid mixtures were used to identify fatty acid peaks. Results are expressed as the HS-Omega-3 Index^®^, which is the sum of EPA and DHA as a percent of total fatty acids in erythrocyte membrane [32], and calculated using a sliding correction factor. The coefficient of variation for the HS-Omega-3 Index^®^ is approximately 5% [33].

### 2.6. Secondary Outcome Measures

#### 2.6.1. Executive Function

The Early Years Toolbox tasks Go/No-Go and Mr Ant were used to measure executive function [31]. The Go/No-Go task is a measure of inhibition which is assessed by an impulse control score generated by the accuracy of both the “go” and “no go” trials [31]. The Mr Ant task is a measure of working memory and consists of eight levels of increasing complexity with three trials in each level. Working memory capacity is measured through an indexed score dependent on the number of accurately completed trials in each level by the child [31].

The Behaviour Rating Inventory of Executive Function—Preschool Version (BRIEF-P) is a measure of executive function completed by the parent/caregiver [34]. The BRIEF-P yields five clinical scales of executive function: Inhibit (control impulsivity); Shift (be flexible and move from one situation or activity to the next); Emotional Control (the ability to modulate emotional responses); Working Memory (the process of holding information in mind for the purposes of completing a task/staying with an activity); and Plan/Organise (the ability to anticipate future events, to set goals, and to develop steps ahead of time to complete tasks) [34]. The BRIEF-P also derives four Index and Composite scales (Inhibitory Self-Control; Flexibility; Emergent Metacognition; and Global Executive Composite) and has two validity scales—Inconsistency and Negativity [34].

#### 2.6.2. Electroencephalographic (EEG) Measures

EEG recording was conducted using a Neurosky^®^ MindWave Mobile 2^TM^ headset, a wireless, single-channel, dry-sensor device. Recording was completed during three phases: resting eyes open, resting eyes closed, and active during the Go/No-Go task with a duration of 30 s^−1^ min per task. The EEG data were recorded on a PC via Bluetooth and later processed to quantify the data to provide power in the delta, theta, alpha, and beta EEG bands. From this data, measures of nervous system arousal, resting activation, and task-related activation were calculated to determine whether these changed throughout the study. EEG recording from this device has previously been shown to be a valid reliable measure [35], which can differentiate between children with ADHD and healthy controls [36] and detect the effects of working memory and inhibitory control training in children with ADHD [37,38].

#### 2.6.3. ADHD

ADHD symptoms were measured as a proxy for self-regulation, using the Conners’ Teaching Rating Scale (CTRS-15) [39]. The 15-item questionnaire, completed by parent/caregivers, provided three subscales: Hyperactivity/Impulsivity, Inattention, and Oppositional Behaviour [39].

### 2.7. Control Measures

#### 2.7.1. PUFA Intake

An online validated Polyunsaturated Fatty Acid Food Frequency Questionnaire (PUFA FFQ) [40] was completed by parents/caregivers on behalf of their child to provide an estimate of the child’s PUFA intake. The parents/caregivers were provided with a link to the questionnaire and instructions on how to complete it. The questionnaire contains 38 items which ask about usual dietary habits over 3 months. The fatty acid database used by the PUFA FFQ is based on analytical data (i.e., foods that have been analysed for their fatty acid content) [41] and the PUFA FFQ automatically calculates the actual PUFA intakes.

#### 2.7.2. Compliance to Study Protocol and Assessment of Blinding of Treatment Groups

Although the main measure of adherence to the study intervention was the HS-Omega-3 Index^®^, given that not all children would provide a finger prick sample a self-check calendar was provided to families to record when a dose was consumed, refused, or missed. These were returned at the end of the intervention and the total dose consumed was calculated. Unused sachets were also returned at the end of the intervention to be counted.

At the final clinic visit parents/caregivers were provided with an end of trial questionnaire to assess their experience with the trial, including the question “which treatment do you think your child was assigned to” (omega-3, placebo or don’t know). These responses were collated and compared to the child’s treatment allocation to determine how many participants correctly guessed their allocation per group.

### 2.8. Statistical Analysis

Data were analysed using SPSS Version 25 (IBM Corp, Armonk, NY, USA). For the baseline data, Spearman correlation analysis was conducted to determine if there was relationship between the HS-omega-3 Index^®^ and any of the outcome variables at baseline. Mann–Whitney test was used to determine whether there was a difference in age between groups and Chi-squared test was used to test whether gender differed between groups.

Linear or generalised linear models were used to determine the effect of omega-3 supplementation on all study outcomes depending on the distribution of the outcome variable. Co-variates of age, gender, EEG activation and baseline HS-omega-3 Index^®^ were included in the model. Both intention-to-treat and per protocol analyses was conducted. Significance levels were adjusted according to the number of variables per hypothesis: *p* < 0.025 for self-regulation and *p* < 0.0125 for executive function.

The dietary intake of EPA and DHA (mg/day) estimated from the PUFA FFQ was log transformed to obtain a normal distribution and an independent samples *t* test was used to compare intake between groups.

Chi-Square tests were used to determine whether there was a difference between groups in parent/caregivers guess of treatment allocation (Omega-3, Placebo or don’t know) and whether they were correct or incorrect (excluding the don’t know group).

### 2.9. Sample Size

There were no studies that examined omega-3 supplementation for self-regulation in this age group. Based on calculations from within group changes from a behavioural intervention, we anticipated a between group effect size of 0.3 (Cohen’s *d*) [42]. Adjusting for an intra class correlation of 0.01, with 80% power, alpha of 0.05 and a cluster size of 10, 180 children would be required per group, with 360 in total. However, as this study was a pilot feasibility trial, and based on available resources, we aimed to recruit up to 80 children.

## 3. Results

### 3.1. Study Population

**Figure 1:**
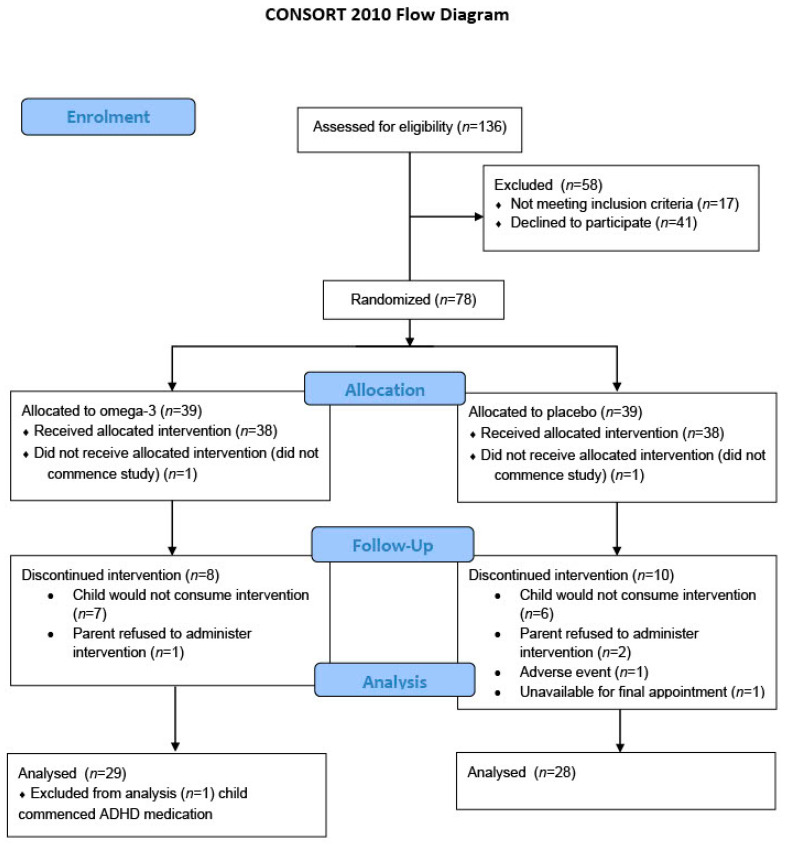
CONSORT diagram.

The families of 136 children expressed interest in the study. From these, 78 were randomised to either the omega-3 or the placebo group with 1 child in each group not commencing the study. From the 38 in each group who commenced treatment, 8 withdrew in the omega-3 group and 10 withdrew in the placebo group. Thirty children in the omega-3 group completed the intervention, however 29 were included in the analysis as one child commenced medication for a behavioural condition midway through the trial and was removed from the final analysis but was able to be included in previous feasibility analysis [28]. In the placebo group, 28 children completed the intervention and were included in the analysis. The reasons for participant withdrawal are outlined in Figure 1.

There was no difference in the distribution of gender between groups with both groups randomly assigned 20 boys and 18 girls. The median age (25th–75th percentile) for the omega-3 group was 4.0 (3.5–4.8) years and months and for the placebo group was 4.4 (3.6–5.1) years and months with no significant difference between groups (*p* = 0.175) (Table 1). Three adverse events were reported (gastrointestinal issues); two in the omega-3 group and one in the placebo group. Only the adverse event in the placebo group resulted in withdrawal from the trial, while the other two participants continued in the trial.

### 3.2. Outcome Measures at 12 Weeks Post-Intervention

**Figure 2:**
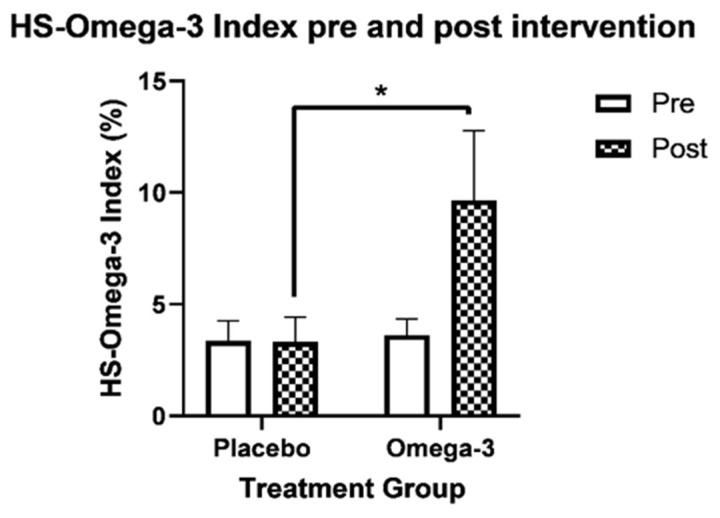
Change in HS-Omega-3 Index^®^ per treatment group, * denotes a significant difference between treatment groups at post-intervention, *p* < 0.001.

At 12 weeks post intervention, there was a significant increase in the HS-Omega-3 Index^®^ in the omega-3 group compared to placebo (*p* < 0.001), as shown in Figure 2. The remaining effects on outcome variables are outlined in Table 1 and Table 2, indicating no other significant change in outcome variables, either from the intention to treat or per protocol analysis for the omega-3 group. However, in the placebo group, behavioural self-regulation on the CSBQ was significantly higher at 12 weeks compared to the omega-3 group.

### 3.3. Omega-3 Index and Outcome Variables at Baseline

**Figure 3:**
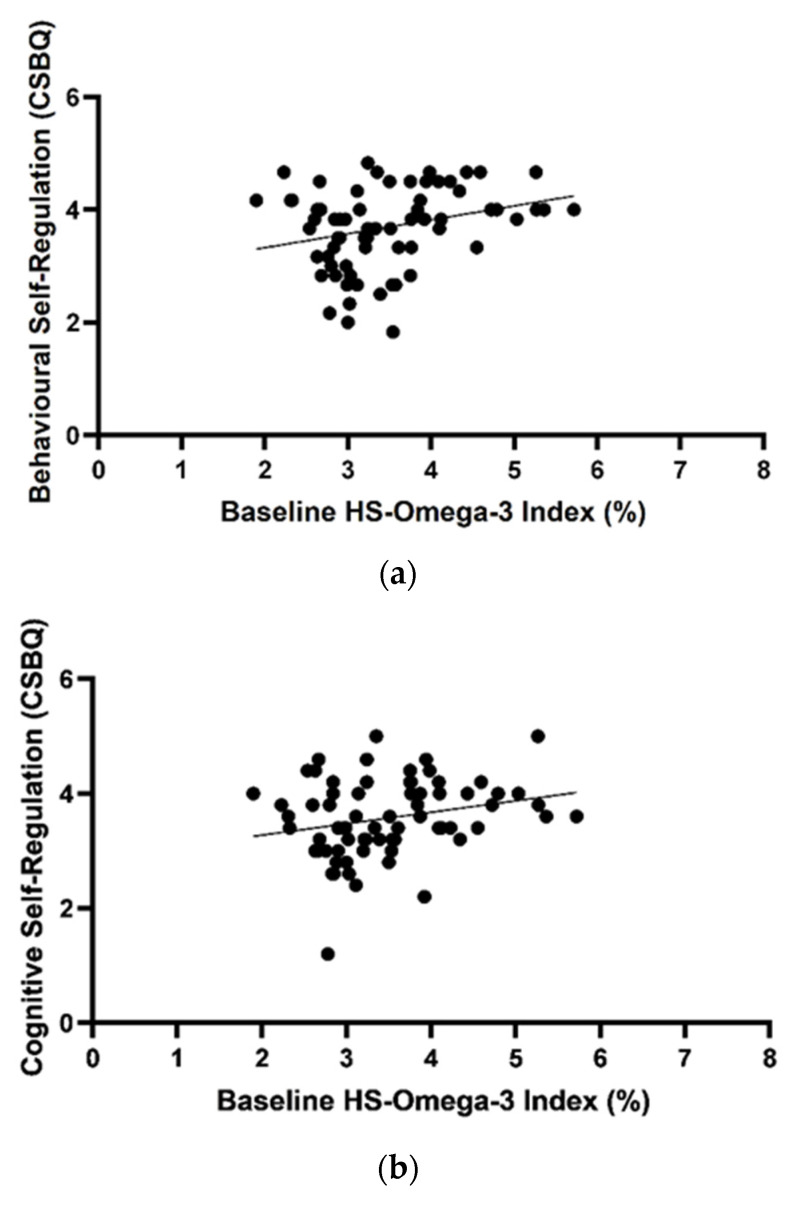

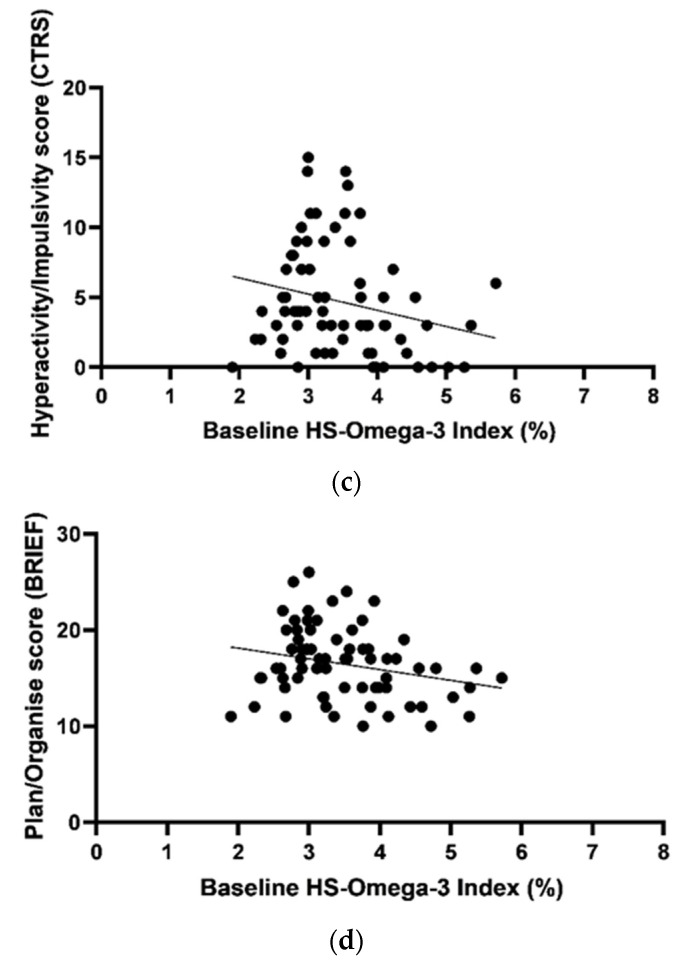
Correlations between the HS-Omega-3 Index^®^ (%) and Behavioural Self-Regulation from the CSBQ (r = 0.287, *p* = 0.015) (**a**), Cognitive Self-Regulation from the CSBQ (r = 0.242, *p* = 0.042) (**b**), Hyperactivity/Impulsivity scale from the CTRS (r = −0.250, *p* = 0.037) (**c**), Plan/Organise raw score from the BRIEF (r = −0.225, *p* = 0.061) (**d**) determined by Spearman correlation.

The relationship between the HS-Omega-3 Index^®^ and all outcome measures at baseline were assessed by Spearman correlations. Significant modest correlations were found between the HS-Omega-3 Index^®^ and the CSBQ Behavioural SR scale (r = 0.287, *p* = 0.015), the CSBQ Cognitive SR (r = 0.242, *p* = 0.042) and the CTRS Hyperactivity/Impulsivity scale (r = −0.250, *p* = 0.037). Furthermore, a trend between HS-Omega-3 Index^®^ and the BRIEF raw score for plan/organise scale (r = −0.225, *p* = 0.061) was detected (Figure 3).

### 3.4. Early Years Toolbox Task Baseline Participant Scores

The proportion of children at baseline who scored within the Early Year’s Toolbox task norms in each quintile for age and gender are presented in Table 3.

### 3.5. Dietary Intake of n-3 LCPUFA

The estimated median dietary intake of EPA and DHA combined determined from the PUFA FFQ was 118 mg/day (IQR 132) for the n-3 LCPUFA group and 104 mg/day (IQR 178) for the placebo group. There was no significant difference in EPA and DHA intake between the two groups determined by an independent samples *t* test (*p* = 0.959).

### 3.6. Blinding of Omega-3 and Placebo

At the end of the trial, parents/caregivers were asked which treatment they thought their child was allocated to during the trial. In the omega-3 group, 53% said they thought their child was on omega-3, 27% thought they were in the placebo group and 20% said they did not know. In the placebo group, 50% said they thought their child was in the placebo group, 21% thought they were in the omega-3 group and 29% said they didn’t know. The distribution of these responses did differ significantly between the groups determined by Chi Square test (*p* = 0.041). When comparing those who guessed correctly and those who guessed incorrectly (removing those who did not know) there was no significant difference between groups (*p* = 0.952). Reported observed benefits from parents whose children were on the omega-3 treatment included “improved concentration”, “improved mood”, “more attentive”.

## 4. Discussion

In this study, n-3 LCPUFA supplementation showed no significant effect on self-regulation in preschool-aged children, although it did result in a nearly three-fold increase in the HS-Omega-3 Index^®^. This significant increase in blood levels of n-3 LCPUFA may be due to the dose of n-3 LCPUFA used in this study which was at the higher end of the scale of previous n-3 LCPUFA intervention studies in children. It has been suggested that increasing the HS-Omega-3 Index^®^ to >6% is more likely to result in cognitive benefit [43]. In the present study, the dose of 1.6g of combined EPA and DHA for 12 weeks was effective in increasing the HS-Omega-3 Index^®^ by almost 3-fold (i.e., 9.7%), suggesting that HiDHA powders are highly bioavailable. This may be due to the microencapsulation of the tuna oil, which reduces oxidation and the small surface area of the particles which are more easily accessible by enzymes allowing greater solubility and absorption [44]. A recent systematic literature review has suggested that the microencapsulated form of omega-3 are more bioavailable compared to standard fish oil [45].

Despite this increase in n-3 LCPUFA blood levels, there were no treatment effects found for the n-3 LCPUFA supplementation. The absence of treatment effects may have been influenced by a number of factors. First, due to the pilot nature of this study, a small sample size was used and therefore most likely our study was underpowered to show an effect. Second, when comparing the scores of the children enrolled in the study to the early years toolbox tasks (Go-No Go, Mr Ant and CSBQ) norms for age and gender, the distribution of participants were skewed to the top three quintiles, suggesting that our study cohort was on average performing higher than “normal” at baseline. This may have led to ceiling effects that, given an under-powered study, impacted upon significance.

While self-regulation and executive functions are complex processes and are influenced by factors such as sleep problems, family financial hardship, angry parenting and gross motor and pre-academic skills, there is also evidence to support the use of n-3 LCPUFA to support the development of self-regulation [46]. However, data from this study and the literature suggest that children who are most likely to benefit from n-3 LCPUFA supplementation are either experiencing clinical disorders and/or underperforming compared to same aged peers. For example, children within the 20th percentile of reading ability improved in behaviour and reading ability after supplementation with DHA [47] and children with higher ADHD scores (9–10 years) responded to n-3 LCPUFA supplementation with improvements reported to visual analysis time, reading speed and phonologic decoding time when compared to children with lower ADHD scores [48]. Furthermore, a recent meta-analysis by Emery et al. (2020) explored the effect of n-3 LCPUFA intervention on cognitive tests in youths, concluding that clinical groups rather than non-clinical groups have benefitted more in domains of working memory, shifting and flexibility, and problem solving [49].

Compared to age and gender norms for the self-regulation test CSBQ, our sample was only below these norms on the emotional self-regulation scale. There was an insignificant but trending effect in the per protocol analysis (0.099) for emotional self-regulation. In a larger better powered study this observation may be important given that improving emotional self-regulation prior to commencing school has been shown to be associated with school functioning in primary school [50]. Furthermore, emotional self-regulation at preschool age drives behavioural self-regulation rather than a relationship in the opposite direction [51].

Baseline levels of n-3 LCPUFA detected in blood samples confirmed expectations of low levels of n-3 LCPUFA in both groups. We did detect a significant relationship between n-3 LCPUFA levels at baseline and measures of self-regulation, executive function, and ADHD symptoms supporting the notion that children with lower levels of erythrocyte omega-3 have poorer self-regulation and executive function. A meta-analysis by Cooper et al. (2015) suggested that children and adults who have lower levels of n-3 LCPUFA may benefit most from n-3 LCPUFA supplementation, which is supported by our findings at baseline [52].

In addition, although 12 weeks of n-3 LCPUFA supplementation was sufficient to see an increase in the HS-Omega-3 Index^®^ a longer intervention duration may have yielded different results. A 12-week intervention period of n-3 LCPUFA supplementation has previously shown positive cognitive and behavioural effects in children in previous studies [48,53,54,55]. However, a longer intervention in this study may have resulted in a significant treatment effect.

The strengths of this study include: (1) a protocol that was found to be feasible and acceptable by participating families [28]; (2) the use of a highly bioavailable novel omega-3 supplement; (3) successful blinding of the treatment with no difference between groups for parents correctly guessing their child’s treatment allocation; (4) the use of validated outcome measures such as the PUFA FFQ; (5) and a specific focus on self-regulation, with previous studies in this age group focussing more broadly on cognitive functions [22,23,24]. Another strength is measuring HS-Omega-3 Index^®^ as recommended by the International Study for the Fatty Acids and Lipids (ISSFAL) Official Statement Number 6: the importance of measuring blood omega-3 long chain polyunsaturated fatty acid levels in research [56].

Limitations of this study include a small sample size due to the pilot nature of the work, which was likely underpowered to detect any significant changes, and a bias within the sample toward higher performance at baseline (ceiling effects). Given the nature of the study cohort, missing data for some variables were also common, for example, some EEG measures had a completion rate of 87–90% as participants had difficulty either wearing the headset or sitting still for the data collection. Lastly, the large number of outcome variables results in multiple comparisons which can mask a true treatment effect.

In conclusion, this pilot study found no significant effect of omega-3 supplementation on self-regulation or executive function in preschool aged children. However, given the baseline relationship between the HS-Omega-3 Index^®^ and outcomes of self-regulation, a larger adequately powered clinical trial is warranted, targeting children who may have suboptimal self-regulation or executive function.

## Figures and Tables

**Table 1:** Primary outcome variables at baseline and post-intervention for the Omega-3 and Placebo Group.

	Baseline Omega-3 Group	Post Intervention Omega-3 Group	Baseline Placebo Group	Post Intervention Placebo Group	Mean Difference	Effect Size (η_p_^2^)	*p*	*p* Adjusted for Age, Gender, EEG	Compliant Only (n Omega-3 Group/Placebo Group)
Age	4.0 (3.5–4.8)		4.4 (3.6–5.1)				0.175		
Gender	20 M, 18 F		20 M, 18 F				1.00		
HTKS	*n* = 26		*n* = 25						
HTKS	15.23 (16.53)	21.04 (18.99)	16.29 (16.93)	26.61 (20.83)	−4.58 (−11.34, 2.19)	0.035	0.181	0.189	0.115 (n = 17/23)
CSBQ	*n* = 29		*n* = 28						
CSBQ Behavioural SR	3.76 (0.63)	3.64 (0.81)	3.99 (0.71)	4.03 (0.91)	−0.347 (−0.60, −0.10)	0.125	0.007	0.018	0.013 (n = 20/23)
CSBQ Cognitive SR	3.59 (0.56)	3.52 (0.62)	3.59 (0.81)	3.67 (0.78)	−0.15 (−0.43, 0.13)	0.022	0.279	0.429	0.465 (n = 20/23)
CSBQ Emotional SR	3.30 (0.78)	3.57 (0.78)	3.40 (0.71)	3.49 (0.64)	0.14 (−0.12, 0.40)	0.022	0.285	0.278	0.099 (n = 20/23)
HS-Omega-3 Index^®^	*n* = 25		*n* = 25						
HS-Omega-3 Index^®^	3.45 (0.54)	9.65 (3.12)	3.31 (0.98)	3.32 (1.12)	6.16 (4.96, 7.38)	0.689	<0.001	<0.001	<0.001 (n = 17/20)

Age presented as median (25th and 75th percentile) years and months. Remaining data presented as mean (SD), change data presented as mean change (95% confidence interval of the difference). M, male; F, female; HTKS, Head Toes Knees Shoulders task; CSBQ, Child Self-Regulation and Social Behaviour Questionnaire; SR, Self-regulation.

**Table 2:** Secondary outcome variables at baseline and post-intervention for the Omega-3 and Placebo Group.

	Baseline Omega-3 Group	Post Intervention Omega-3 Group	Baseline Placebo	Post Intervention Placebo	Mean Difference	Effect Size (η_p_^2^)	*p*	*p* Adjusted for Age, Gender, EEG	Compliant Only
iPad Tasks	*n* = 27		*n* = 28						
Mr Ant	1.63 (0.69)	1.59 (0.72)	1.67 (0.86)	1.86 (0.83)	−0.242 (−0.621, 0.136)	0.031	0.205	0.472	0.270 (n = 19/23)
Go No Go	0.49 (0.24)	0.56 (0.21)	0.63 (0.24)	0.65 (0.19)	0.022 (−0.079, 0.122)	0.004	0.664	0.635	0.990
BRIEF *	*n* = 27		*n* = 27						
BRIEF Inhibit	26.63 (5.75)	25.37 (7.47)	25.00 (5.25)	24.07 (6.25)	−0.38 (−2.56, 1.80)	0.002	0.725	0.960	0.734
BRIEF Shift	14.96 (3.59)	14.56 (3.33)	14.52 (3.68)	14.41 (3.85)	−0.158 (−1.64, 1.33)	0.001	0.831	0.807	0.779
BRIEF emotional control	16.89 (3.42)	16.22 (4.56)	16.37 (3.64)	16.63 (3.33)	0.178 (−1.38, 1.74)	0.001	0.820	0.588	0.573
BRIEF working memory	26.89 (6.67)	25.70 (7.12)	24.93 (5.55)	23.22 (6.35)	0.77 (−1.52, 3.05)	0.009	0.504	0.617	0.665
BRIEF Plan/organise	16.44 (3.95)	15.81 (4.14)	15.96 (3.56)	14.89 (3.70)	0.526 (−0.84, 1.89)	0.012	0.444	0.209	0.624
BRIEF ISCI	43.52 (8.24)	41.59 (10.68)	41.37 (7.44)	39.70 (8.81)	−0.26 (−3.51, 2.98)	0.001	0.870	0.803	0.637
BRIEF FI	31.85 (5.93)	30.78 (6.64)	30.89 (6.17)	30.04 (6.29)	0.01 (−2.50, 2.51)	<0.001	0.997	0.843	0.880
BRIEF EMI	43.33 (10.22)	41.52 (10.93)	40.89 (8.60)	38.11 (9.61)	1.21 (−2.02, 4.43)	0.011	0.456	0.412	0.598
BRIEF GEC	101.81 (18.59)	97.67 (21.63)	96.78 (16.72)	92.22 (19.35)	0.76 (−6.08, 7.60)	0.001	0.825	0.663	0.941 (n = 19/22)
EEG measures ^¥^	*n* = 24		*n* = 23						(n = 18/19)
EEG EC theta/beta	12.34 (7.22)	10.64 (6.65)	11.54 (4.78)	10.19 (3.39)	0.28 (−2.80, 3.35)	0.001	0.857	0.768	0.825
(EC-EO) Delta (24/22)	51.61 (68.54)	17.43 (69.87)	61.69 (81.57)	20.39 (34.62)	−1.39 (−33.71, 30.94)	<0.001	0.932	0.756	0.265
(EC-EO) Theta	38.99 (45.03)	23.59 (39.98)	36.76 (31.61)	21.91 (27.01)	2.38 (−18.11, 22.87)	0.001	0.816	0.884	0.417
(EC-EO) Alpha	−1.14 (8.19)	−0.59 (6.31)	3.15 (7.87)	1.17 (6.71)	−1.59 (−5.50, 2.32)	0.014	0.428	0.515	0.672
(EC-EO) Beta	−6.29 (13.14)	−2.74 (6.57)	−2.68 (5.70)	−3.82 (5.00)	1.72 (−1.66, 5.10)	0.023	0.310	0.371	0.525
CTRS	*n* = 29		*n* = 28					n = 26/27	n = 20/23
CTRS-15 Hyperactivity/Impulsivity	4.79 (3.90)	4.83 (4.38)	4.00 (3.06)	4.18 (3.28)	0.002 (−1.41, 1.41)	<0.001	0.998	0.896	0.767
CTRS-15 Inattention	4.66 (3.73)	4.55 (3.68)	3.14 (3.14)	3.29 (2.87)	0.214 (−1.11, 1.54)	0.002	0.748	0.766	0.537
CTRS-15 Opposition	4.66 (2.88)	3.90 (2.47)	3.61 (2.51)	3.54 (2.80)	−0.40 (−1.36, 0.56)	0.013	0.406	0.330	0.188

Data presented as mean (SD), change data presented as mean change (95% confidence interval of the difference). * Brief adjusted for gender and EEG only; ^¥^ EEG adjusted for age and gender only; BRIEF, Behaviour Rating Inventory of Executive Function; ISCI, Inhibitory Self-Control Index; FI, Flexibility Index; EMI, Emergent Metacognition Index; GEC, Global Executive Composite; EEG, Electroencephalographic; EC, eyes closed; EO, Eyes open; CTRS, Conners Teachers Rating Scale.

**Table 3:** Children’s performance of Early Years Toolbox tasks at baseline.

	CSBQ Behavioural SR	CSBQ Cognitive SR	CSBQ Emotional SR	Go No-Go iPad Task	Mr Ant iPad Task
Quintile	Number (%)	Number (%)	Number (%)	Number (%)	Number (%)
1	9 (11.8)	10 (13.2)	21 (28.0)	13 (18.1)	11 (14.9)
2	10 (13.2)	7 (9.2)	16 (21.3)	14 (19.4)	12 (16.2)
3	25 (32.9)	28 (36.8)	12 (16.0)	16 (22.2)	10 (13.5)
4	17 (22.4)	7 (9.2)	6 (8.0)	18 (25.0)	30 (40.5)
5	15 (19.7)	24 (31.6)	20 (26.7)	11 (15.3)	11 (14.9)
Total	76 (100)	76 (100)	75 (100)	72 (100)	74 (100)

Data presented is number of children (%) per quintile. CSBQ, Child Self-Regulation and Social Behaviour Questionnaire; SR, Self-regulation.

## Data Availability

The data presented in this study are available on request from the corresponding author.

## Supplementary Materials

The following are available online at https://www.mdpi.com/article/10.3390/nu13103561/s1, Table S1: CONSORT checklist.
